# Improving Atomic Force Microscopy Imaging by a Direct Inverse Asymmetric PI Hysteresis Model

**DOI:** 10.3390/s150203409

**Published:** 2015-02-03

**Authors:** Dong Wang, Peng Yu, Feifei Wang, Ho-Yin Chan, Lei Zhou, Zaili Dong, Lianqing Liu, Wen Jung Li

**Affiliations:** 1 State Key Laboratory of Robotics, Shenyang Institute of Automation, Chinese Academy of Sciences, Shenyang 110016, China; E-Mails: wangdong@sia.cn (D.W.); yupeng@sia.cn (P.Y.); wangfeifei@sia.cn (F.W.); zhoulei@sia.cn (L.Z.); dzl@sia.cn (Z.D.); 2 University of Chinese Academy of Sciences, Beijing 100049, China; 3 Department of Mechanical and Biomedical Engineering, City University of Hong Kong, Kowloon, Hong Kong, China; E-Mail: hoyin.chan@gmail.com

**Keywords:** atomic force microscope, hysteresis, piezoelectric actuator, direct inverse asymmetric PI model, feedforward control

## Abstract

A modified Prandtl–Ishlinskii (PI) model, referred to as a direct inverse asymmetric PI (DIAPI) model in this paper, was implemented to reduce the displacement error between a predicted model and the actual trajectory of a piezoelectric actuator which is commonly found in AFM systems. Due to the nonlinearity of the piezoelectric actuator, the standard symmetric PI model cannot precisely describe the asymmetric motion of the actuator. In order to improve the accuracy of AFM scans, two series of slope parameters were introduced in the PI model to describe both the voltage-increase-loop (trace) and voltage-decrease-loop (retrace). A feedforward controller based on the DIAPI model was implemented to compensate hysteresis. Performance of the DIAPI model and the feedforward controller were validated by scanning micro-lenses and standard silicon grating using a custom-built AFM.

## 1. Introduction

The atomic force microscope (AFM) is a powerful tool for nanoscale imaging and manipulation [1,2]. In an AFM, piezoelectric materials are often used as nanopositioning actuators to drive a scanning probe because of their high resolution capabilities and fast response time [3]. However, the inherent nonlinearities in piezoelectric actuators, especially the hysteresis [4], will lead to displacement errors in horizontal direction in AFM scanning images [5].

Several methods have been proposed to compensate the hysteresis effect. They can be generally classified into two categories: (1) feedback control; and (2) model-based feedforward control. Feedback control method is known to have modeling errors that are simpler to handle, and they have errors caused by parameter variations that can be minimized [6]. However, the hysteresis effect can make a feedback control system unstable [7]. As for model-based feedforward control, it improves performance without incurring the stability problems associated with feedback design. However, the challenges associated with model-based feedforward control are model accuracy and computational complexity [8].

**Figure 1.**
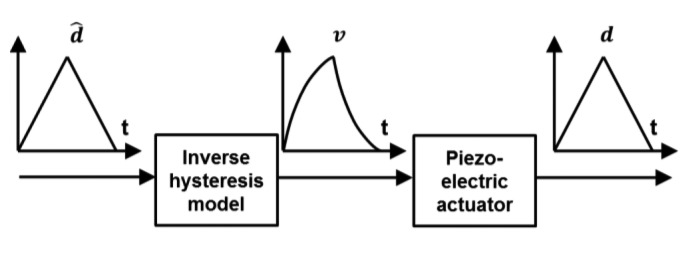
Illustration of the operating principle of a model-based feedforward controller. Through the inverse PI model, a desired linear displacement *d̂(i)* is transformed into an non-linear voltage *v(i)*. The piezoelectric actuator travels on a linear trajectory *d(i)* when driven by *v(i)*.

**Figure 2.**
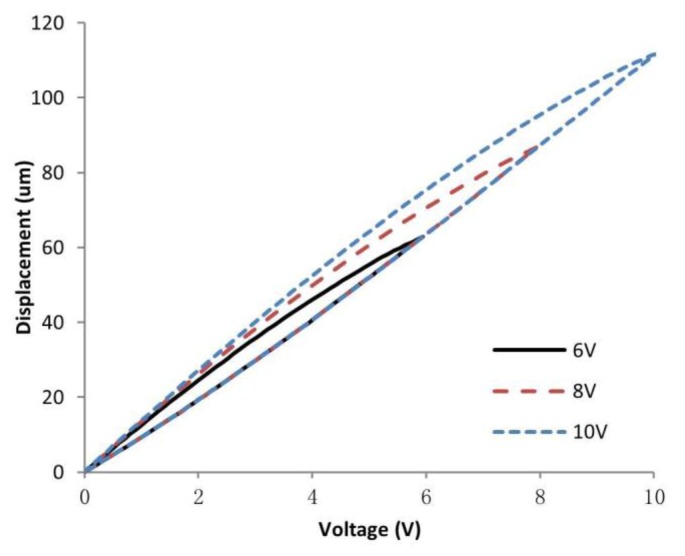
Asymmetric hysteresis loops of a piezoelectric actuator under different driving peak voltages. The hysteresis effect becomes more obvious with increasing driving peak voltages.

The keys to successfully develop a feedforward control include: (1) develop a hysteresis model which is close to the real hysteresis curve; and (2) realize a feedforward controller based on an inverse model to linearize the response of the actuators, as illustrated in Figure 1. The most commonly used feedforward models include Maxwell's slip model [9], Duhem model [10], Krasnosel'skii-Pokrovskii operator [11], Preisach model [12] and Prandtl-Ishlinskii (PI) model [13]. Among all these hysteresis models, the PI model is the most suitable for real-time applications, such as AFM real-time scanning. This is because it has a much simpler implementation procedure and it also has a unique analytical inverse model [14,15]. However, feedforward controller based on conventional symmetric PI (SPI) model will lead to inevitable error when compensating asymmetric hysteresis of piezoelectric actuators which have asymmetric voltage-displacement response (see Figure 2).

In order to compensate asymmetric hysteresis more accurately, some feedforward controllers based on modified PI (MPI) hysteresis models have been investigated, such as adding new components to the conventional SPI model [3,16], using a generalized play operator for characterizing asymmetric hysteresis nonlinearity [17], and using different width parameters in trace and retrace branches [18]. Among these MPI models, the one proposed in [18] is easier to implement because the model does not involve operator modifications and extra components. However, it is central-symmetric and cannot describe the asymmetric hysteresis as shown in Figure 2.

**Figure 3.**
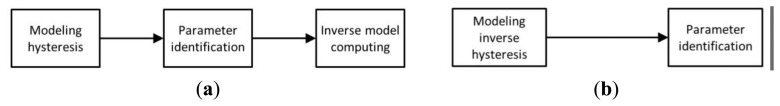
Procedure of establishing inverse hysteresis model: (**a**) the conventional method; (**b**) the modified method.

To characterize and compensate asymmetric hysteresis without increasing model complexity, we describe in this paper our work on developing an inverse asymmetric PI (API) hysteresis model with different slope parameters in trace and retrace branches. In order to reduce computation, instead of establishing an inverse model using conventional three-step method (see Figure 3a), a two-step method proposed in [19] by Qin, *et al.*, which can model inverse hysteresis directly, was used. We will present in this paper the experimental results from implementing this DIAPI hysteresis model and the related feedforward controller in a custom-built AFM.

Our team has previously demonstrated in 2011 that an asymmetric PI model gives more accurate AFM images than a symmetric model [5]. In the current work, we made the following revisions and extensions to the prior work: (1) based on the work of Qin, *et al.* [19], the direct inverse modeling process is used instead of computing an inverse model based on a forward model; (2) in order to illustrate the performance of API model in AFM imaging, a normalized square *L_2_* norm (*NSL2*) that describes dissimilarity was used instead of the normalized product correlation that describes similarity; (3) a micro-lens AFM-scan experiment was added to further evaluate the performance of the direct inverse modeling process, *i.e.*, see Section 3.3; (4) in order to illustrate the asymmetric hysteresis of the piezoelectric actuator, the difference between the slope parameters is shown in this paper.

The rest of the paper is organized as follows: Section 2 presents the principle of the DIAPI model and characterization of parameters; Section 3 discusses the experimental results to interpret the advantages of using the DIAPI model, which include less error and less computational requirement; finally, our conclusions are presented in Section 4.

## 2. Symmetric & Asymmetric PI Inverse Models

In this section, both SPI and DIAPI models used in this study are presented.

### 2.1. Definition of Backlash Operator

**Figure 4.**
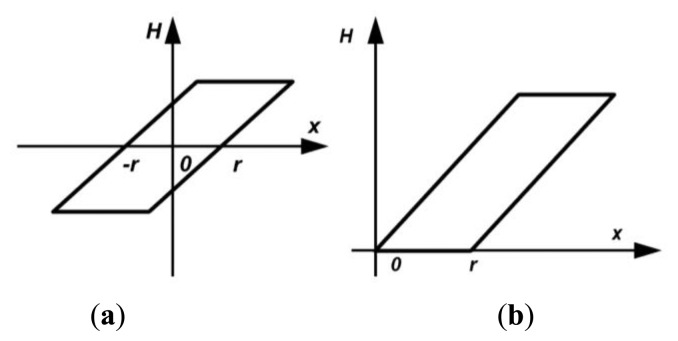
Backlash operators: (**a**) play operator; (**b**) OSP operator.

In this study, forward hysteresis model means the mapping of driving voltage to actuator displacement and inverse hysteresis model means the mapping of actuator displacement to driving voltage. Both of these PI models have same form which is a phenomenological model composed of many elementary rate-independent symmetric backlash operators. Backlash operator can be expressed in two forms: (1) play operator [3] (see Figure 4a); (2) one side play (OSP) operator [16] (see Figure 4b). Since the input voltage of the piezoelectric actuator in our custom-built AFM is positive, the OSP operator is preferred.

### 2.2. Modeling of Hysteresis by Conventional PI Inverse Model

According to the definition in [16], the conventional SPI inverse model can be expressed by:

(1) $$v\left(i\right)=\vec{\omega }\vec{H}=\sum_{j=1}^{N}\omega \left(j\right)H\left(i,j\right)$$

(2) H(i,j)=max{d(i)−r(j),min[d(i),H(i−1)]})

where *v* is the output of the PI model (and represents the input voltage of the piezoelectric actuator), *H* is an OSP operator, *N* is the number of backlash operators, *ω* is the slope of the backlash (*ω* = 1 means a 45° slope), *d* is the input of the SPI inverse model (and represents the displacement of the piezoelectric actuator), and *r* is width of the backlash. This type of SPI inverse model uses a single slope to describe both the trace and retrace section, which will lead to intrinsic errors when describing an asymmetric hysteresis induced by a piezoelectric actuator as shown in Figure 2.

### 2.3. Modeling of Hysteresis by DIAPI Model

In order to solve the problem mentioned, we established a DIAPI inverse model that use separate slope parameters to describe trace and retrace branches respectively. The DIAPI inverse model is defined by:

(3) $$v_{a}\left(i\right)=\vec{m}{\vec{\omega }}_{a}\vec{H}\left(i\right)=\vec{m}{\left[\begin{array}{cc} {\vec{\omega }}_{af} & {\vec{\omega }}_{ab} \end{array}\right]}^{T}\vec{H}\left(i\right)$$

where *m⃗* = [1 0] or *m⃗* = [0 1] for trace and retrace section, respectively. Two slope parameters *ω⃗_af_* and *ω⃗_ab_* are used to describe trace and retrace branches, respectively. The initial condition of the backlash operator is defined by:

(4) $$H\left(0\right)=\max\left\{d\left(0\right)-r,\min\left[d\left(0\right)+r,h_{0}\right]\right\}$$

where *h_0_* is set to 0 if the piezoelectric actuator starts from its de-energized state.

In this DIAPI inverse model, three parameters have to be determined: (1) number of backlash operators *N*; (2) width parameters *r⃗*; and (3) slope parameters *ω⃗_af_* and *ω⃗_ab_*. The number of backlash operator *N* is set to be 10 (as discussed in [3]). The width parameters *r⃗* are given by:

(5) $$\begin{array}{cc} r_{i}=\frac{i-1}{N}\left[\max\left(d\right)-\min\left(d\right)\right], & i=1\cdots N \end{array}$$

The slope values *ω⃗_af_* and *ω⃗_ab_* can be determined by minimizing the following error function of a least-squares fit method:

(6) $$E\left(\omega \right)=\sum_{i}^{m}\left[v_{a}\left(i\right)-v_{\text{actual}}{\left(i\right)}^{2}\right]$$

where *v_actual_(i)* is the control voltage of piezoelectric actuator.

## 3. Experimental Results

A custom-built AFM using piezoelectric actuators was built and experiments were performed using this system to validate the DIAPI hysteresis model and the corresponding model-based feedforward controller.

### 3.1. Experimental Setup

**Figure 5.**
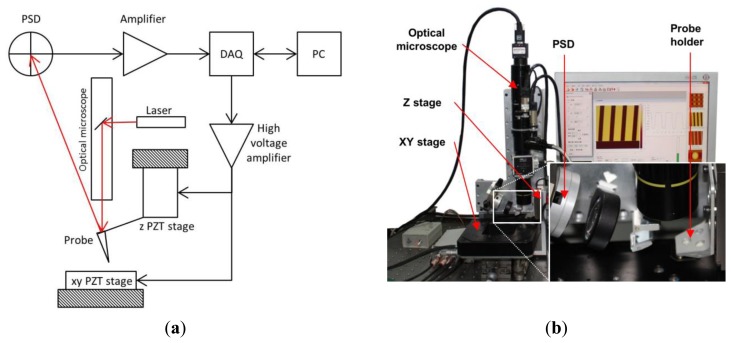
A custom-built AFM system. (**a**) Schematic view of the custom-built AFM; (**b**) AFM scan head.

A schematic view of the custom-built AFM is shown in Figure 5a. The AFM system mainly consists of two parts: a controller and a scan head module (see Figure 5b). The controller contains a PC, two DAQ cards and a three-channel high voltage amplifier. The scan head module (shown in Figure 5b) consists of two nano-positioning stages, a probe holder, an optical microscope, a two-dimension position sensitive device (PSD, an optical position sensor that can measure the position of a light spot in one or two-dimension on a sensor surface) and mirrors. A nanopositioning stage with a 12 μm travel range and a sensitivity of 0.6 μm/V is used as the actuator in vertical (Z-axis) direction. A nano-positioning stage, with a travel range of 100 μm × 100 μm and a sensitivity of 10 μm/V, is used as the actuator in horizontal (XY) plane. The displacement of the nano-positioning stage in the XY direction is measured by a capacitive sensor (CS05, Micro-Epsilon Messtechnik GmbH&Co. KG, Ortenburg, Germany), which has a measurement range of 0∼500 μm and a dynamic resolution of 10 nm at an 8.5 kHz sampling frequency. The surrounding temperature of the experimental setup is maintained at 20 °C.

### 3.2. Model Prediction Experiment

The DIAPI model is used to model the inverse hysteresis of the nanopositioning stage in the X and Y directions, e.g., horizontal direction. As the modeling steps and results are the same in both directions, only those in X direction are shown. Considering AFM commonly works in raster scan mode in the horizontal direction with low scan speed (commonly less than 10 Hz), the hysteresis loop can be approximated as rate-independent. Therefore, the nanopositioning stage is excited using a 1 Hz triangular waveform input with various amplitudes and the displacement was measured by the CS05 capacitive sensor with 1 KSPS sampling rate. The hysteresis loop with 100 μm displacement that was excited by 9.1 V amplitude input voltage was used for parameter identification.

According to the algorithm presented in Section 2, the parameters of the DIAPI model have been calculated and are listed in Table 1. To illustrate asymmetry of hysteresis, difference between slope parameters in trace and retrace branches is calculated by:

(7) $$e_{\omega }=\left|\frac{\omega_{af}-\omega_{ab}}{\omega_{ab}}\right|$$

and shown in Table 1. It can be seen that the difference between two slope parameters can be large (at most 90%). Therefore, modeling error of SPI model will be inevitably large if the same slope parameters used in trace and retrace branches.

**Figure 6.**
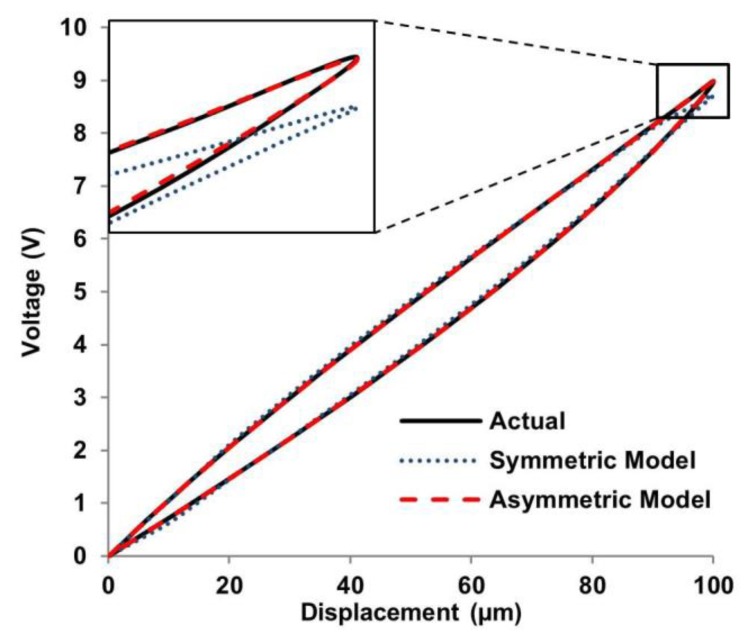
Actual hysteresis loop and PI model loops.

**Figure 7.**
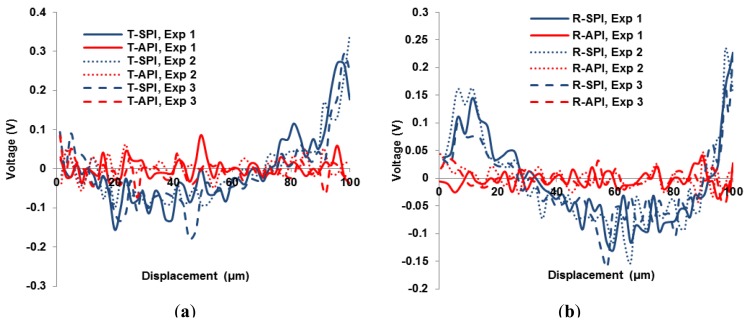
(**a**) Trace and (**b**) retrace errors of the SPI and DIAPI models are labeled as T-SPI and T-API, respectively. Ten experiments were performed to show the consistency of experimental results. Three representative data sets are shown in this figure.

Experimental results are shown in Figures 6 and 7. In Figure 6, errors between the SPI prediction and the actual hysteresis loop are larger than predicted by the DIAPI model. Ten experiments were performed and showed consistent results. Three representative data sets of the modeling errors in trace and retrace branches from the two models are shown in Figure 7a and Figure 7b, respectively. A smaller error means the corresponding model is better in predicting the hysteresis. From Figure 7a,b, it can be seen that errors of the DIAPI model are obviously smaller than that of SPI model especially in range of 20 μm∼60 μm, 90 μm∼100 μm during trace and 0 μm∼20 μm, 50 μm∼80 μm, 90 μm∼100 μm during retrace. The RMS and maximum errors of the SPI and DIAPI model in 10 experiments are listed in Table 2. The mean RMS errors of SPI model and DIAPI model are 0.08 V (with s.d. of 0.003) and 0.02 V (with s.d. of 0.002), respectively. The mean RMS error of DIAPI model is 75% smaller than that of the SPI model.

**Figure 8.**
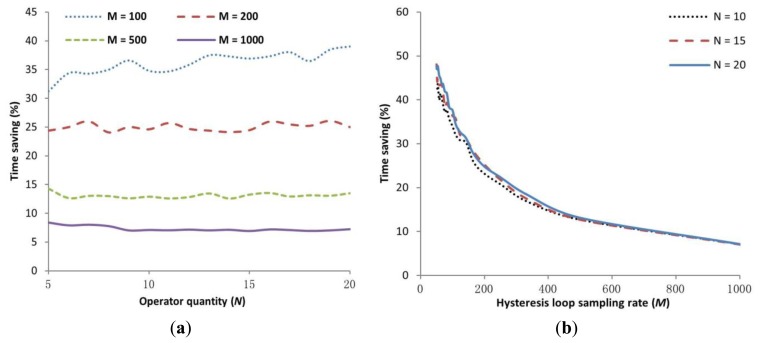
Time saving percentage of DIAPI model over IIAPI model with (**a**) various hysteresis loop sampling rate and (**b**) operator quantity.

Compared to conventional three-step methods that are used in indirect inverse API (IIAPI) models, the DIAPI model uses less computational time because the inverse model computing step is eliminated. In order to quantitatively compare the computational time consumption of the two models, IIAPI and DIAPI models were tested with various hysteresis loop sampling rate (*M*) and operator quantity (*N*). The IIAPI model from [5] is used for this comparison. The identification and computation algorithms were coded in Matlab, which was installed in a PC with an i5 CPU and 8 G RAM. To reduce the effect of contingency, the algorithms were run 10,000 times for each model and the mean run-time was used for comparison. The time-saving percentages of DIAPI model over IIAPI model are shown in Figure 8.

As shown in Figure 8a, the time-saving percentage fluctuates slightly when the quantity of *N* increases. Also, as shown in Figure 8b, the time-saving percentage decreases when *M* increases, *i.e.*, when more samples are used in a hysteresis loop. According to experiments, both models are more accurate when *M* increases, but the accuracy does not further improve much when *M* exceeds 200. Hence, referring to Figure 8b, we can conclude that DIAPI model can save ∼25% (*M* = 200) more run-time than the IIAPI model.

### 3.3. AFM Imaging of Micro-Lenses

**Figure 9.**
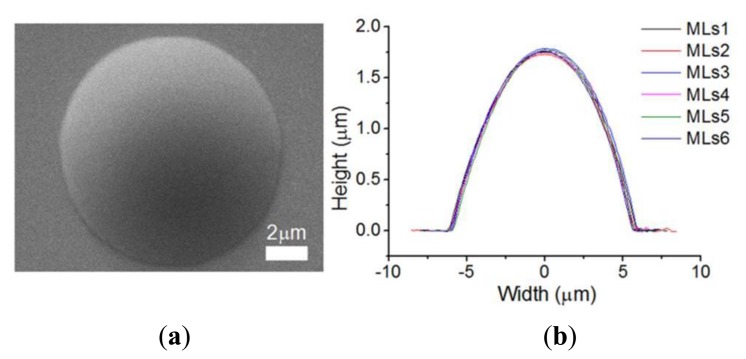
(**a**) An SEM image of a PDMS micro-lens; (**b**) The section curves of PDMS micro-lenses scanned by Bruker AFM. MLs 1-6 represent section curves of six micro-lenses.

**Figure 10.**
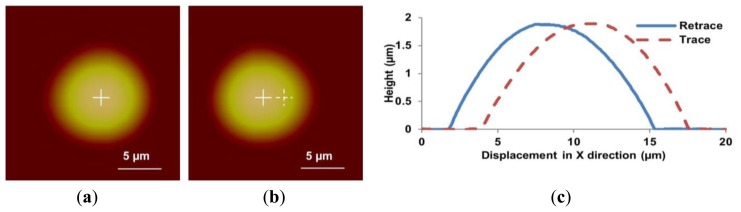
Height images of a PDMS micro-lens scanned using the custom-built AFM without calibration. (**a**–**c**) are trace image, retrace image and cross-section curves, respectively. The white cross represents location of the micro-lens center. In the retraced image, the dashed white cross represents the location of the micro-lens center in the trace image. The center of the micro-lens is shifted about 2.5 μm because of hysteresis.

To demonstrate the improved performance of the DIAPI model in AFM imaging, spherical micro-lenses which are made of Polydimethylsiloxane (PDMS) were used and scanned in contact mode. The type of AFM probe used is MLCT which is manufactured by Bruker (Santa Barbara, CA, USA). The micro-lenses were first scanned using SEM and a commercial AFM (Dimension 3100, Bruker) using feedforward calibration (closed loop off) as a reference. The SEM image and section curves are shown in Figure 9a,b. Next, in order to illustrate image distortion caused by hysteresis of the piezoelectric stage, the micro-lens was scanned by the custom-built AFM without calibration. As shown in Figure 10a,b, the location of the micro-lenses in trace and retrace image are shifted because of the hysteresis of the piezoelectric stage. Detailed differences can be observed in cross-section curves (see Figure 10c), e.g., trace curve and retrace curves do not overlap because of hysteresis.

**Figure 11.**
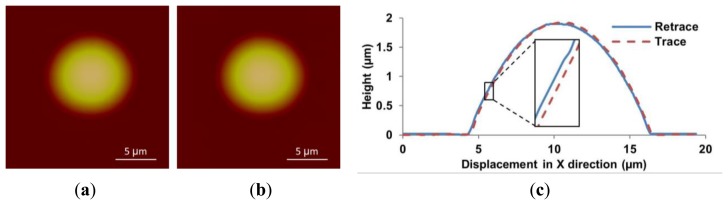
Height images of the PDMS micro-lens scanned using the custom-built AFM with the SPI model calibration. (**a**–**c**) are trace image, retrace image and cross-section curves, respectively. Inset shows a 5× magnified view of cross-section between 5.4 μm and 6 μm.

**Figure 12.**
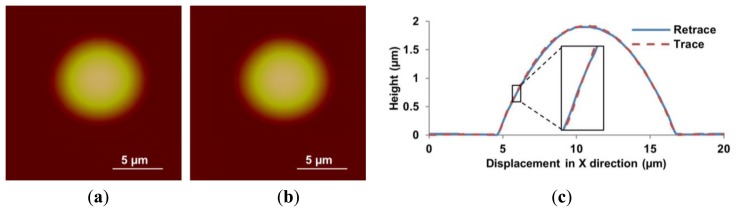
Height images of PDMS micro-lens scanned using the custom-built AFM with DIAPI model calibration. (**a**–**c**) are trace image, retrace image and cross-section curves, respectively. Inset shows a 5× magnified view of the cross-section between 5.4 μm and 6 μm.

**Figure 13.**
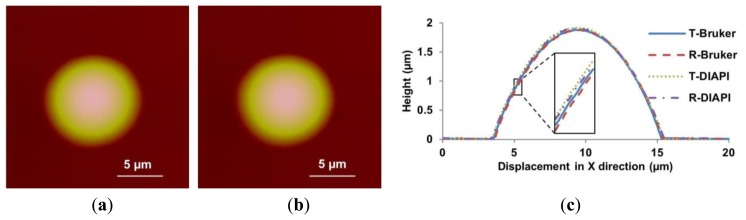
Height images of PDMS micro-lens scanned using the Bruker AFM. (**a**,**b**) are trace and retrace images scanned by the Bruker AFM, respectively; (**c**) shows the cross-section curves scanned by the Bruker AFM and the custom-built AFM based on DIAPI model. T-Bruker and R-Bruker represent trace and retrace cross-section curves scanned using the Bruker AFM, respectively; T-DIAPI and R-DIAPI represent trace and retrace cross-section curves using the custom-built AFM based on DIAPI model, respectively. Inset shows a 5× magnified view of the cross-section between 5.1 μm and 5.7 μm.

The feedforward controller based on the DIAPI model was implemented on custom-built AFM for hysteresis calibration. The parameters of inverse model were calculated using Equations (5) and (6). For comparison, the feedforward controller based on SPI model was also implemented. Figures 10, 11 and 12 show height images and cross-section curves of micro-lenses scanned using the custom-built AFM based on feedforward controller using SPI and DIAPI models and Bruker AFM, respectively. As can be seen from height image (Figures 11a,b and 12a,b) of micro-lenses, distortion of the image was corrected. The trace and retrace images become visually identical. Furthermore, the cross-section curves based on DIAPI model (see Figure 12c) coincide better than that of Bruker AFM (see Figure 13c), while the one based on SPI model (see Figure 11c) coincide the worst.

In order to quantitatively compare the difference between AFM images based on DIAPI and SPI models, normalized square *L_2_* norm (*NSL2*) describing dissimilarity between trace and retrace images can be given by [20]:

(8) $$\begin{array}{c} D=\overset{n}{\underset{i=1}{\text{∑}}}{\left(\frac{x_{i}-\bar{x}}{\sigma_{x}}-\frac{y_{i}-\bar{y}}{\sigma_{y}}\right)}^{2}\\ \sigma_{x}=\sqrt{\frac{1}{n}\overset{n}{\underset{i=1}{\text{∑}}}{\left(x_{i}-\bar{x}\right)}^{2}},\sigma_{y}=\sqrt{\frac{1}{n}\overset{n}{\underset{i=1}{\text{∑}}}{\left(y_{i}-\bar{y}\right)}^{2}},\bar{x}=\frac{1}{n}\overset{n}{\underset{i=1}{\text{∑}}}x_{i},\bar{y}=\frac{1}{n}\overset{n}{\underset{i=1}{\text{∑}}}y_{i} \end{array}$$

where *D* represents *NSL2, i.e.*, dissimilarity between two images, the smaller the better; *x_i_* and *y_i_* are values of each pixel in trace and retrace images after binarization, respectively. The threshold of binarization is the mean gray value of image. To illustrate the asymmetry of hysteresis, the difference between the *NSL2* parameter in trace and retrace images is calculated by:

(9) $$e_{D}=\frac{D_{S}-D_{A}}{D_{S}}$$

Table 3 summarizes the *NSL2* between the trace and retrace images of the micro-lenses scanned by the custom-built AFM and Bruker AFM, respectively. *D_N_, D_S_, D_A_* and *D_B_* represent the *NSL2* without calibration, SPI model calibration, DIAPI model calibration, and Bruker AFM feedforward calibration, respectively. Without calibration, it is obvious that the location of the micro-lens in the trace image is different from that in the retrace image (see Figure 10a,b). The mean *NSL2* of 10 experiments is 45,814. After the model-based feedforward controllers were implemented, the trace and retrace images had no major image shift and the means *NSL2* of 10 experiments dropped to 2322 (SPI model) and 1292 (DIAPI model). As reference, the mean *NSL2* of 10 experiments scanned using the Bruker AFM was 1545. Hence, we can conclude that the PI models will definitely improve AFM imaging consistency during the trace and retrace steps. The *NSL2* between the trace and retrace images based on DIAPI model is 44.3% smaller than that of SPI model and 16.3% ((*i.e.*, (*D_B_* − *D_A_)/D_B_*) smaller than that of Bruker AFM with feedforward calibration.

### 3.4. AFM Imaging of Silicon Grating

**Figure 14.**
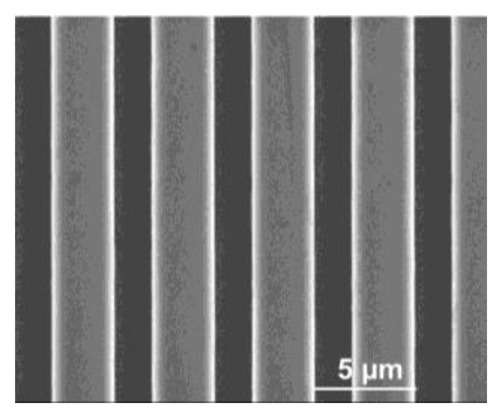
A SEM image of a silicon grating.

**Figure 15.**
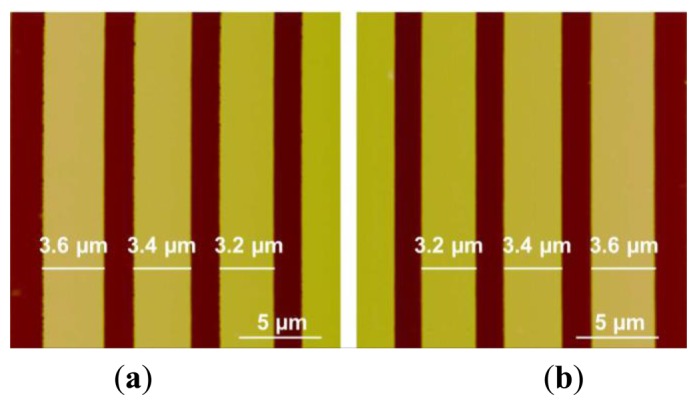
Height images of pitches in silicon grating scanned using custom-built AFM without calibration. (**a**) Trace image; (**b**) retrace image.

**Figure 16.**
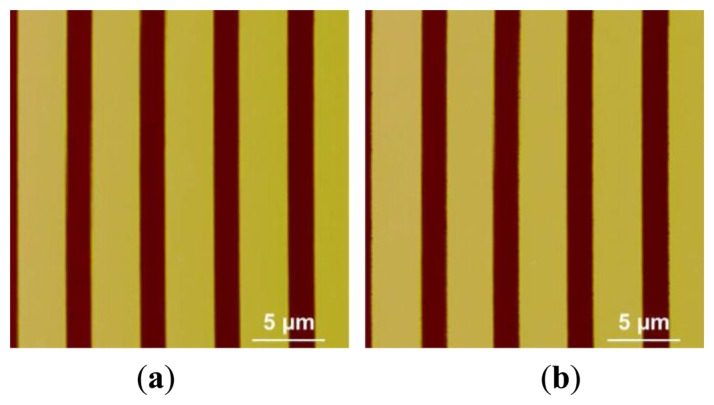
Height images of pitches in silicon grating scanned using the custom-built AFM with SPI model calibration. (**a**) Trace image; (**b**) retrace image.

**Figure 17.**
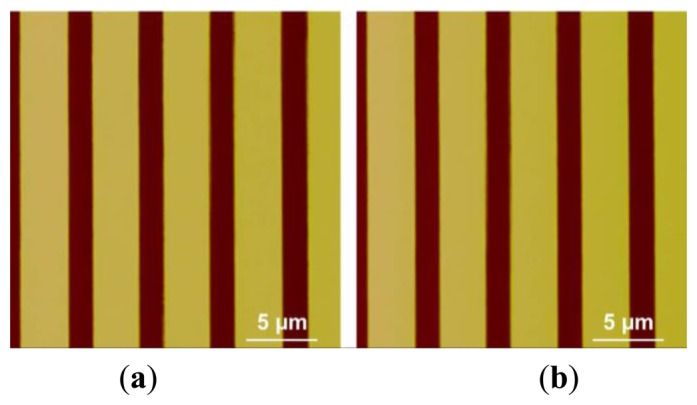
Height images of pitches in silicon grating scanned using the custom-built AFM with DIAPI model calibration. (**a**) Trace image; (**b**) retrace image.

**Figure 18.**
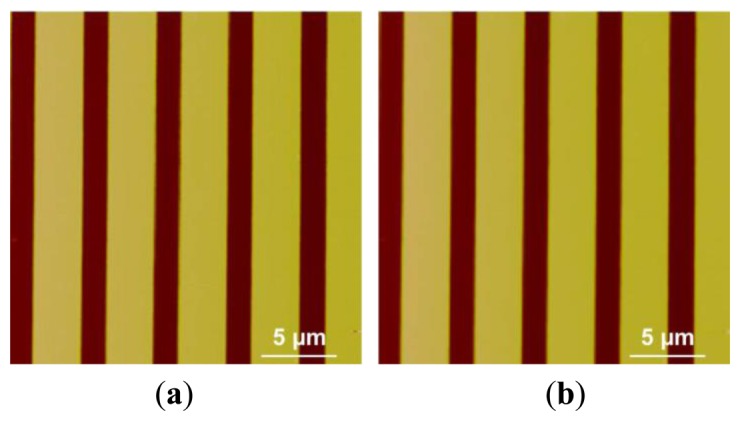
Height images of pitches in silicon grating scanned using the Bruker AFM. (**a**) Trace image; (**b**) retrace image.

Besides scanning the micro-lens, a silicon grating (HS-500MG, Innovative Solutions, Sofia, Bulgaria) with pitches (500 nm high and 5 μm period) was also scanned using the custom-built AFM under same machine settings of that used in micro-lens scan. The profiles of the pitches are shown in SEM image (Figure 14). The trace and retrace images of the pitches without calibration are shown in Figure 15. We can see that pitches of trace image and retrace image were shifted. In trace image, the pitch (3.6 μm) on the left is wider than that on the right (3.2 μm). In retrace image, the pitch (3.2 μm) on the left is narrower than that on the right (3.6 μm). Figures 16 and 17 are images of the pitches after implementing the SPI and DIAPI models, respectively. We can see from the figures that trace and retrace image become visually equal after calibration. In order to further confirm the improvement of AFM imaging based on DIAPI model, silicon grating images scanned using the Bruker AFM are shown in Figure 18.

Table 4 summarizes the *NSL2* between trace and retrace images of the pitches scanned by the custom-built AFM based on the SPI and DIAPI models. Without calibration (*i.e.*, PI model implementation), mean *NSL2* of 10 experiments is ∼178,272. After model-based feedforward controllers were implemented, the mean *NSL2* dropped to 6467 (SPI model) and 4023 (DIAPI model). As reference, the mean *NSL2* of 10 experiments scanned using the Bruker AFM was 4717. Hence, there is a 37.8% improvement of the DIAPI model over the SPI model, and a 14.7% (*i.e.*, (*D_B_-D_A_)/D_B_*) improvement over the Bruker AFM.

It should be noted that the hysteresis of piezoelectric actuators is less significant in a small travel range, *i.e.*, asymmetric hysteresis is less significant for small scan range of an AFM. Therefore, when the scan range decreases, the difference between the SPI and DIAPI models becomes smaller, and the *NSL2* difference between the trace and retrace images based on SPI and DIAPI models also become smaller. Hence, the advantage of the DIAPI model over the SPI model is less obvious when the AFM scan range is smaller.

## 4. Conclusions

In this paper, an asymmetric PI model using two series slope parameters in trace and retrace branches is proposed to model the inverse asymmetric hysteresis of a piezoelectric actuator. The parameters of the inverse model were identified by a least-square fit instead of computation based on the loading curve. A feedforward controller based on the inverse DIAPI model was implemented on a custom-built AFM for validation. Model prediction experiment shows that mean RMS error of DIAPI model is 75% smaller than that of the SPI model, and the establishment of the DIAPI model is 25% faster than that of the IIAPI model. We have shown experimentally that both the SPI and DIAPI models are effective in improving the precision of the resulting AFM scanned images. Furthermore, the DIAPI model yields significantly lower *NSL2* values between the trace and retrace scanned images, e.g., 44.3% and 37.8% lower in micro-lenses and silicon grating scanning experiments, respectively.

## Figures and Tables

**Table 1.** Recognized parameters of the DIAPI model.

**i**	**r(i)**	**ω_af_ (i)**	**ω_ab_ (i)**	*_e_*_*ω*_
1	0	0.1068	0.1274	16%
2	11.1	−0.0080	−0.0204	61%
3	22.2	−0.0046	−0.0094	51%
4	33.3	−0.0034	−0.0066	48%
5	44.4	−0.0034	−0.0049	31%
6	55.5	−0.0008	−0.0040	80%
7	66.6	−0.0027	−0.0036	25%
8	77.7	−0.0003	−0.0030	90%
9	88.8	−0.0011	−0.0010	10%
10	99.9	−0.0012	−0.0025	52%

**Table 2.** Errors of SPI and DIAPI model.

**Date Set**	**RMS Errors (V)**		**Maximum Errors (V)**	

	**SPI Model**	**DIAPI Model**	**SPI Model**	**DIAPI Model**
1	0.078	0.021	0.260	0.068
2	0.083	0.019	0.277	0.062
3	0.082	0.021	0.297	0.054
4	0.079	0.023	0.293	0.061
5	0.073	0.021	0.283	0.064
6	0.080	0.024	0.287	0.068
7	0.085	0.022	0.284	0.067
8	0.081	0.023	0.271	0.056
9	0.081	0.021	0.263	0.064
10	0.076	0.020	0.259	0.065
Mean	0.080	0.021	0.277	0.063
σ	0.003	0.002	0.013	0.005

**Table 3.** *NSL2* between trace and retrace image of micro-lenses.

**Number**	***D****_N_* **(Without Calibration)**	***D****_S_* **(SPI Model)**	***D****_A_* **(DIAPI Model)**	***E****_D_* ***(*%*)***	***D****_B_* **(Bruker AFM)**
1	45,887	2011	1136	43.5	1404
2	46,104	2674	1042	61.0	1302
3	46,091	2481	1096	55.8	1898
4	47,938	2570	1336	48.0	1778
5	45,711	2563	1539	40.0	1883
6	43,865	1908	1399	26.7	1400
7	45,833	1924	1308	32.0	1298
8	43,024	2543	1106	56.5	1390
9	47,135	2679	1318	50.8	1453
10	46,556	1868	1649	11.7	1648
Mean	45,814	2322	1293	44.3	1545
σ	1436.3	346.0	200.7		235.3

**Table 4.** *NSL2* between trace and retrace image of pitches.

**Number**	***D****_N_* **(Without Calibration)**	***D****_S_* **(SPI Model)**	***D****_A_* **(DIAPI Model)**	***E****_D_* **(%)**	***D****_B_* **(Bruker AFM)**
1	177,690	6474	4066	37.2	4890
2	180,457	6804	3772	44.6	4359
3	169,440	6184	3781	38.9	4451
4	185,401	6707	3869	42.3	4413
5	180,865	6675	4343	34.9	5176
6	182,269	6037	3866	36.0	4876
7	175,774	5981	4321	27.8	4771
8	180,452	6472	3857	40.4	4971
9	171,847	7069	4415	37.5	5007
10	178,529	6267	3944	37.1	4251
Mean	178,272	6467	4023	37.8	4717
σ	4818.7	352.5	247.3		320.9
